# Supplementary material for: Crawling and Gliding: A Computational Model for Shape-Driven Cell Migration
Source: PLoS Comput Biol. 2015 Oct 21;11(10):e1004280. doi: 10.1371/journal.pcbi.1004280 (PMC4619082; doi:10.1371/journal.pcbi.1004280)
Supplement: S1 Code — (ZIP) [file pcbi.1004280.s012.zip › release/tst/doc/html/crash_8h.html]

Tissue Simulation Toolkit: crash.h File Reference


Tissue Simulation Toolkit
 0.1.4.1


- Main Page
- Namespaces
- Classes
- Files

- File List
- File Members

Functions

crash.h File Reference

This graph shows which files directly or indirectly include this file:

Go to the source code of this file.

|  |  |
| --- | --- |
| Functions | |
| void | StartSIGINTHandling () |
|  | |
| void | HandleSIGINT (int dummy) |
|  | |
| void | StartSIGSEGVHandling () |
|  | |
| void | HandleSIGSEGV (int dummy) |
|  | |
| void | NiceMessage () |
|  | |
| void | MemoryWarning (void) |
|  | |
| void | Crash (char \*message) |
|  | |

## Function Documentation

|  |  |  |  |  |  |
| --- | --- | --- | --- | --- | --- |
| void Crash | ( | char \* | *message* | ) |  |

Referenced by HandleSIGINT(), HandleSIGSEGV(), and MemoryWarning().

|  |  |  |  |  |  |
| --- | --- | --- | --- | --- | --- |
| void HandleSIGINT | ( | int | *dummy* | ) |  |

References Crash().

Referenced by StartSIGINTHandling().

|  |  |  |  |  |  |
| --- | --- | --- | --- | --- | --- |
| void HandleSIGSEGV | ( | int | *dummy* | ) |  |

References Crash().

Referenced by StartSIGSEGVHandling().

|  |  |  |  |  |  |
| --- | --- | --- | --- | --- | --- |
| void MemoryWarning | ( | void |  | ) |  |

References Crash().

Referenced by CellularPotts::AllocateSigma(), PDE::AllocateSigma(), CellularPotts::FindCellDirections(), CellularPotts::GrowInCells(), CellularPotts::ReadZygotePicture(), and CellularPotts::SearchNandPlot().

|  |  |  |  |  |
| --- | --- | --- | --- | --- |
| void NiceMessage | ( |  | ) |  |

Referenced by StartSIGINTHandling().

|  |  |  |  |  |
| --- | --- | --- | --- | --- |
| void StartSIGINTHandling | ( |  | ) |  |

References HandleSIGINT(), and NiceMessage().

|  |  |  |  |  |
| --- | --- | --- | --- | --- |
| void StartSIGSEGVHandling | ( |  | ) |  |

References HandleSIGSEGV().


---

Generated on Thu Aug 14 2014 22:04:01 for Tissue Simulation Toolkit by  

 1.8.6
